# Reply to Engzell: Maybe in plain sight but out of focus

**DOI:** 10.1073/pnas.2219213120

**Published:** 2023-01-03

**Authors:** Nate Breznau, Eike Mark Rinke

**Affiliations:** ^a^SOCIUM Research Center on Inequality and Social Policy, University of Bremen, Bremen 28334, Germany; ^b^School of Politics and International Studies, University of Leeds, Leeds LS2 9JT, United Kingdom

We thank Engzell (1) for making points about our study (2) that are both important independent of our findings and valuable as additional perspectives on the evidence reported. Overall, Engzell reminds us about careful hypothesis specification, measurement, and causal inference. These are things coming to the fore in recent methodological discussions (3–5), and with which we wholeheartedly agree and see as key takeaways from our main findings.

As a reminder, the main point of the research reported in our article was neither to identify the best possible test for a given hypothesis nor to conduct a replication of previous findings. It also was not to identify if any particular teams successfully used the data to test causality—although being nonexperimental in nature certainly does not exclude data from the potential to test causal claims (6, 7). Instead, our study held potential sources of variance in research design constant at levels that emulate a situation commonly encountered in the social sciences: a given hypothesis and data suspected to be appropriate for testing it. Then, when different teams were free to take their own approaches to this hypothesis, we observed the variation in findings and analytical decisions that followed.

This resulting variation was broad and remained after adjusting for all observed model components including teams’ potential usage of up to eight different measures drawn from the data as input variables predicting variance in their numerical outcomes and subjective conclusions. In this process, we identified that between-team variance was 11.2% of total variance, which is not only a nontrivial proportion (8) but also the basis for broad disagreement in the subjective conclusions of the teams. As we report in the article, “13.5% (12 of 89) of the team conclusions were that the hypothesis was not testable given these data, 60.7% (54 of 89) were that the hypothesis should be rejected, and 28.5% (23 of 89) were that the hypothesis was supported.” Such between-team variation is important because a single subjective conclusion is usually a headline takeaway of a given scientific publication. Therefore, our study demonstrates that any single study’s outcomes might differ and should be interpreted as a draw from an unobserved distribution whose shape and weights are mostly unknown or unappreciated, often only inferred from simulations.

Moving forward, we believe the scientific community has much to learn in the interpretation of interresearcher and intermodel variance, although we recognize that some important first steps have been taken (9, 10). Engzell’s letter coupled with our findings provide a reminder that it will be an important task for future many-analyst studies to examine more systematically the degree to which greater specificity in theory and hypotheses, identification of estimands, attention to measurement, and use of causal inference might reduce analytical flexibility and variation in scientific results. If the reduction is massive, as Engzell presumes, our concerns about the “hidden universe of uncertainty” may have solutions hiding in plain sight. At this time, we lack the evidence required to know whether this is clearly the case.
